# Formation and Characterization of Two Magnetic Three-Dimensional Spheroid Models of Murine Pancreatic Adenocarcinoma

**DOI:** 10.3390/mps8040075

**Published:** 2025-07-07

**Authors:** Magali Perier, Litan Wang, Marine Simonneau, Jacqueline Ngo-Reymond, Julie Guillermet-Guibert, Maxime Lafond, Cyril Lafon

**Affiliations:** 1 LabTAU, INSERM, Centre Léon Bérard, Université Claude Bernard Lyon 1, 69003 Lyon, France; litan.wang@sorbonne-universite.fr (L.W.); marine.simonneau@inserm.fr (M.S.); jacqueline.ngo@inserm.fr (J.N.-R.); maxime.lafond@inserm.fr (M.L.); cyril.lafon@inserm.fr (C.L.); 2 Centre de Recherches en Cancérologie de Toulouse (CRCT), CNRS-Inserm, Université de Toulouse, 2 av Hubert Curien, 31037 Toulouse, France; julie.guillermet@inserm.fr

**Keywords:** spheroid, pancreatic adenocarcinoma, magnetic aggregation

## Abstract

Pancreatic adenocarcinoma remains one of the deadliest cancers, with limited treatment options and high chemoresistance. Traditional 2D cell cultures fail to accurately replicate the tumor architecture. Our study introduces three-dimensional (3D) pancreatic adenocarcinoma spheroid models using magnetic aggregation of pancreatic cancer cells and immortalized fibroblasts in either liquid culture medium or embedded in hydrogels. The spheroids’ growth was characterized using optical imaging, while viability was assessed using ATP quantification and flow cytometry. Results demonstrated successful spheroid formation and growth. Further analysis suggested that on one hand, culture in liquid medium and ATP-based viability assessment are practical for initial experiments. On the other hand, hydrogel culture and flow cytometry, although being more resource- and labor-intensive, provided both a more reproducible and detailed viability analysis.

## 1. Introduction

Pancreatic cancer, one of the most lethal and aggressive cancers, ranks as the 12th most common cancer globally and the sixth leading cause of cancer-related mortality according to the global cancer statistics 2022 [1]. Pancreatic ductal adenocarcinoma (PDAC) is the most common type of pancreatic cancer, accounting for about 85% of pancreatic cancer cases [2]. Currently, surgical resection and adjuvant chemotherapies are the principal treatments of PDAC. However, only primary-stage cancer with non-metastatic diseases can be resected surgically [3]. Chemotherapy is primarily utilized to treat early-stage pancreatic cancer post- surgical resection or in an attempt to downstage a more advanced cancer to the resectability criteria [2]. While various protocols of adjuvant chemotherapies were substantially improved in recent years, drug treatment efficacy remains suboptimal, maintaining the average 5-year survival rate of PDAC below 10% [4].

A contributing factor to drug discovery failure is the inaccuracy of drug response due to the inappropriate models tested in the drug discovery processes [5,6]. These traditional models, including two-dimensional (2D) cell models and animal models, have inherent limitations. The 2D cell models, where cells are cultured as a monolayer, are prevalent for drug screening because of their simplicity and low cost. However, their inability to replicate the interactions between cells or between cells and the extracellular matrix (ECM), and the potential changes in the phenotype of cell lines based on in vitro passage cycles, render them insufficient to represent the multifaceted biological features of pancreatic tumors [7]. Animal models, although instrumental, pose challenges due to species differences, extensive model establishment period, and high experimentation cost, limiting their utility [8]. The primary challenge in chemotherapy lies in the inherent chemoresistance of pancreatic cancer [9], stemming from the unique tumor microenvironment (TME). PDAC’s TME is replete with fibrotic stroma and various cell types, including cancer-associated fibroblast (CAFs), macrophage, inflammatory cells and nerve cells, and ECM components [2,10]. The dense stroma alters the pancreatic tumor’s physical environment, increasing stiffness and hydrostatic pressure, thereby impeding drug uptake [11]. Establishing a PDAC model capable of emulating the pancreatic TME and accurately portraying PDAC features is imperative for achieving accurate drug test responses towards preclinical developments.

Bridging the gap between in vitro and in vivo, 3D cellular models have demonstrated considerable benefits over the past decades [12]. Comprising one or multiple cell lines, these models can reproduce the tumor environment’s complexity and biological features. Studies on PDAC 3D models have demonstrated capability to exhibit high expression of matrix proteins and matrix gene miRNA, dense stroma fibrosis, and hypoxic and necrotic centers [13,14,15].

There are numerous approaches to fabricating 3D models, broadly categorized into scaffold-based and scaffold-free spheroids systems [16]. Scaffold-based models usually employ hydrogels as principal material and act as the natural ECM structure, providing a favorable environment for cells’ growth, proliferation, and migration. However, the scaffold-based models’ systems are challenging in terms of reproducibility due to their complex architecture. Some scaffolds may contain the functional groups capable of undergoing reactions with the tested drug [12]. Contrastingly, scaffold-free systems, appreciated for their simplicity and affordability, rely on the self-assembly of cells in a non-adherent environment. The cellular aggregates, usually referred as spheroids, have their own ECM environment. In pancreatic cancer research, the spheroid fabrication methods is seemingly the most popular due to their relatively simple setups [17]. The magnetic 3D bioprinting technique, one of the most popular scaffold-free methods for spheroids’ generation, has been largely developed and proven suitable for various cell types and applications in disease study, tissue engineering, drug discovery, and screening [18,19,20,21,22]. This technique, renowned by its simplicity and efficiency, leverage magnetic nanoparticles to aggregate cells under a magnetic field. Those magnetic particles are biocompatible and can attach to the plasma membrane electrostatically [23]. This methods facilitates the rapid production of multiple uniform 3D models [24]. Additionally, magnetic loading of the spheroids allows for elasticity characterization [25], which is particularly relevant in studying the mechanical environment in pancreatic cancer.

The spheroids, composed of pancreatic tumor cells and fibroblasts magnetized by magnetic nanoparticles, were tested for their sensitivity to gemcitabine hydrochloride, a standard PDAC chemotherapy agent [26]. After identifying this model’s limitations, we combined the magnetic 3D bioprinting technique with the hydrogel scaffold-based technique to fabricate more viable and sustainable spheroids. Indeed, the magnetic integration bioprinting method can enable biophysical studies that would be impossible without the magnetic field. This feature enabled us to characterize our spheroids using magnetic micro-elastography [25].

Our study may serve as a pivotal reference for studies on PDAC 3D models and may pave the way to optimizing future PDAC 3D models by integrating the magnetic 3D bioprinting technique with the hydrogel scaffold system.

In our study, we present a comprehensive method for generating magnetics spheroids using the magnetic 3D bioprinting technique combined with the use of U-bottom plates to optimize spheroid formation as well as to perform viability assays and drug sensitivity testing. The novelty of our study is to compare different spheroid culture conditions and different viability measurement methods. Using basics methods for characterizing a 3D model, we were able to carry out a comparative analysis of different protocols, each of which has its own advantages and disadvantages, as we demonstrate.

## 2. Materials and Methods

The step-by-step protocols have been published on protocol.io.

Spheroids in culture medium, accessed on 28 March 2025. dx.doi.org/10.17504/protocols.io.3byl4z772vo5/v1.

Spheroids in hydrogel, accessed on 1 April 2025. dx.doi.org/10.17504/protocols.io.261geeoxyg47/v1.

Spheroids by centrifugation, accessed on 3 April 2025. dx.doi.org/10.17504/protocols.io.x54v97m6qg3e/v1.

Viability assay by CellTiter-Glo™ 3D, accessed on 28 March 2025. dx.doi.org/10.17504/protocols.io.5qpvoo55xv4o/v1.

Viability assay by flow cytometry, accessed on 28 March 2025. dx.doi.org/10.17504/protocols.io.5qpvoo5yxv4o/v1.

Viability assay by prestoblue, accessed on 28 March 2025. dx.doi.org/10.17504/protocols.io.3byl4z7k2vo5/v1.

### 2.1. Cells Lines

Tumor spheroids consisted of cancer cells, co-cultured with fibroblasts that produced a collagen meshwork. KPC A219 and iMEF were obtained from the Toulouse Cancer Research Center (CRCT, Team SigDYN, Toulouse, France). The KPC A219 cancer cells, derived from a transgenic mouse, carry pancreas-specific Kras and p53 mutations (Kras^LSL-G12D/+^; Trp53^LSL-R172H/+^; Pdx-1-Cre), which mimics in vivo the most frequent gene mutations found in PDAC [27]. The mouse embryonic fibroblasts (iMEF) were immortalized by pSicoR p53 (pSicoR p53 (Addgene Plasmid #12090) from Dr. Tyler Jacks’s lab) and therefore expressed Enhanced Green Fluorescent Protein (EGFP). The cells were cultured under standard conditions (37 °C, 5% CO_2_) in a Modified Eagle Medium Nutrient Mixture F-12 (DMEM/F-12) (Gibco 21331020, Paisley, UK) supplemented with 10% Fetal Bovine Serum (FBS) (Gibco 10270-106, Paisley, UK), 1% L-glutamine (Gibco 25030-024, Paisley, UK), 1% Penicillin/Streptomycin (Gibco 15140-122, Paisley, UK). The cells were maintained in the 75 cm^2^ T-flask and passaged twice a week.

For 2D sensitivity to gemcitabine, cells were detached from 75 cm^2^ T-flask with trypsin 0.05 EDTA, counted and seeded in 96-well plate one day before adding gemcitabine. The number of cells seeded were 4000 per well for 24 h gemcitabine incubation and 2000 per well for 72 h.

### 2.2. Spheroid Generation

#### 2.2.1. Spheroids in Culture Medium (SphM)

**Figure 1:**
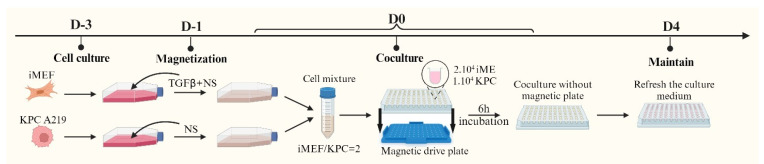
SphM generation process from D-3 to D0.

Nanoshuttles (NS) (Greiner Bio-one 657846, Frickenhausen, Germany) are nanoparticles composed of gold particles (Au), iron oxide (Fe_2_O_3_) and poly-L-lysine. They can be incorporated into the cells without affecting their viability, proliferation, and chemosensitivity [23]. Spheroids can be formed upon the agglomeration of NS-bearing cells using a magnetic field [22] (Figure 1).

KPC A219 and iMEF cells were cultured separately in two flasks with 15 mL of culture medium. The KPC A219 were cultured in 25 cm^2^ T-flasks, starting with 200,000 cells. The iMEF were cultured in 75 cm^2^ T-flasks, starting with 600,000 cells. After two days of incubation, Transforming Growth Factor beta 1 (TGF-β1) (PEPROTECH 100-21, Cranbury, NJ, USA) was added in the iMEF culture solution, reaching a concentration of 50 ng/mL. TGF-β1 can activate iMEF cells, inducing α smooth muscle actin expression (α-SMA) [27]; activated fibroblasts are described as the most enriched cancer-associated fibroblasts in PDAC desmoplasia [28,29]. Then, 100 µL and 150 µL of NS were added to the KPC A219 and iMEF culture flasks, respectively, to compensate the multiplying rate discrepancies between the two cell lines and to obtain the same number of NS per cell at day 3. On the day of co-culture, the cells were trypsinized, counted, and seeded into a U-bottom cell-repellent 96-well plate (Greiner Bio-One 647846, Frickenhausen, Germany). Each well contained 10,000 KPC A219 cells, 20,000 iMEF cells, and 150 µL of culture medium. The plate was then placed on a magnetic drive plate (Greiner Bio-one 655840, Frickenhausen, Germany) capable of producing a magnetic field to aggregate the cells in the center of each well. The magnetic drive plate and the cells were then incubated under standard condition (37 °C, 5% CO_2_). After 6h of incubation, the magnetic drive plate was removed. Following another 4 days of incubation, and then twice a week, the culture medium was replaced with 100 µL of fresh culture medium.

#### 2.2.2. Spheroids in Hydrogel (SphG)

**Figure 2:**
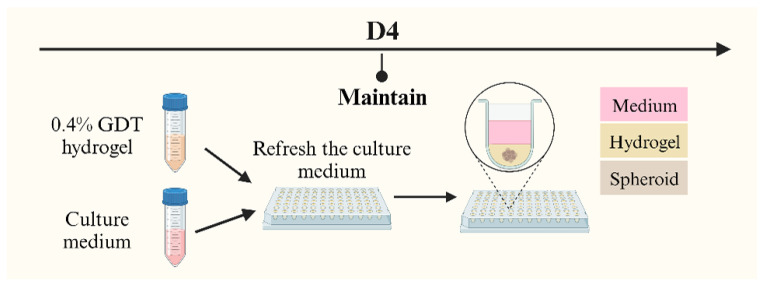
SphG generation process at D4.

The primary distinction between SphM and SphG lies in the culture solution to maintain the spheroids alive. After a 4-day co-culture, the culture medium for SphG was replaced with 100 µL of GrowDex^®^-Transparent (GDT) hydrogel for 3D cell culture (UPM Biomedicals 100103005, Helsinki, Finland) (Figure 2). Unlike traditional culture medium, this cellulose-based, animal component-free GDT hydrogel can emulate the extracellular matrix (ECM) and promote cell growth and proliferation.

The GDT hydrogel was prepared at a concentration of 0.4% volume in the culture medium, with this specific concentration based on its optimization for spheroid growth. The process of applying GDT hydrogel followed the manufacturer’s protocol. Briefly, the existing culture medium was first replaced with 100 µL of the 0.4% GDT hydrogel. An additional 100 µL of the culture medium was then layered on top, and the plates were incubated at 37 °C. The culture medium on top of the gel was replaced by 100 µL of fresh culture medium twice a week. The hydrogel remained at the bottom of the well and was not changed until degradation of the gel.

For spheroid isolation from the hydrogel, GrowDase™ enzyme (GDE) (UPM Biomedicals 900102002, Helsinki, Finland) was employed, as recommended by the GDT manufacturer. GDE was prepared at a concentration of 450 µg/mg of GDE, according to the manufacturer instructions. Each well received 100 µL of GDE. After introducing GDE into each well, it was left to incubate for a minimum of 8 h, overnight. This duration was dictated by the minimal time required for complete GDT hydrogel degradation.

#### 2.2.3. Spheroids by Centrifugation

KPC A219 and iMEF cells were cultured in the same condition as SphM. TGF-β1 was added the day before spheroids formation but not NS. Cells were seeded in a U-bottom cell-repellent 96-well plate with the same number of cells as SphM. Then, the plate was centrifuged 4 min at 300× *g*, at room temperature. The plate was rotated by 180 degrees and centrifuged again for 4 min at 300× *g*.

### 2.3. Characterization of Spheroids

#### 2.3.1. Viability Assay

We investigated two distinct methods to assess the viability of the spheroids: the measurement of adenosine triphosphate (ATP) production and the measurement of live cells in flow cytometry. For the 2D culture, we used PrestoBlue^TM^ HS Cell Viability Reagent (Invitrogen P50200, Eugene, OR, USA) to assess the viability of our cells. CellTiter-Glo™ 3D Cell Assay reagent (Promega G968A, Madison, WI, USA), referred to as CT3D, can be used to measure the quantity of produced ATP and is specifically developed as a proxy for 3D cellular model viability estimation.

The CT3D assay was conducted following the manufacturer’s protocol. The spheroids were first transferred to a 96-well opaque plate (Corning COSTAR 3917). The medium of each spheroid was replaced with 50 µL of DMEM/F-12 medium supplemented just with 1% L-glutamine and 1% Penicillin-Streptomycin (without FBS) and 50 µL of CellTiter-Glo™ 3D Cell Assay solution. The solution mixture was then placed on an orbital plate shaker for 5 min at 200 rpm and incubated at room temperature for 25 min. The luminescence signal of ATP was then measured using a luminometer (TECAN Infinite 200PRO M Plex, Grödig, Austria) in the relative luminescence units (RLU) with 1 s of integration time. For each measurement, an ATP standard curve was established with ATP concentrations of 0, 0.5 µM, 1 µM, 2 µM, and 3 µM for normalizing the luminescent signal data and converting it to ATP concentration.

The effectiveness of the reagents was evaluated using 3D models of different sizes, by varying the initial cell count with a fixed 2-to-1 iMEF-KPCA219 ratio. Initial total cell counts of 3000, 6000, 15,000, and 30,000 cells were evaluated. The viability of the spheroids was measured after an 11-day incubation period (D11). The Celltiter-glo 3D cell viability assay was selected for the subsequent studies. The viability of both SphM and SphG was assessed on D1, D4, D8, D11, and D15.

Flow cytometry-based viability measurements were performed using AM Blue calcein (Invitrogen c1429, Eugene, OR, USA) after spheroid dissociation. Upon entering the cell, intracellular esterases cleave the AM group. Apoptotic and dead cells with compromised cell membranes do not retain calcein. Membrane-permeable live-cell labeling dye is suitable for flow cytometry. For each reading, 8 spheroids were pooled in a 24-well cell-repellent plate. Spheroids were washed with 500 µL of DPBS and subsequently incubated at 37 °C in Accumax (Sigma A7089, Saint-Quentin-Fallavier, France) with low-speed agitation. Spheroids were flushed manually using a 20 µL pipette and low-retention tips approximately every 10 min. After 45 min, a portion of the cells that had already dissociated, corresponding to the outer layers of the spheroids (subsequently referred to as the “periphery”), was collected. The remaining cells were incubated for another 45 min. This dissociation time was determined empirically using microscopy observations. After dissociation, the cells were centrifuged at 300× *g* for 5 min at room temperature and rinsed with 500 µL of DPBS. They were then incubated in 500 µL of calcein at 7 µg/mL for 15 min in the dark. Finally, the cells were centrifuged and resuspended in 300 µL of DPBS. The analysis was performed on a BD Biosciences LSRII flow cytometer (Becton Dickinson, San Jose, CA, USA). Calcein, indicative of viability, was measured using a DAPI laser, and FITC-expressing iMEF cells were identified using an Alexa Fluor 488 laser. For each measurement, controls consisted of iMEFs only, KPC A219 only, iMEFs without calcein, KPC A219 without calcein, and iMEFs + KPC A219 in a 2-to-1 ratio. The flow cytometer viability measurements were carried out on a sample of 10,000 events.

PrestoBlue^TM^ HS Cell Viability Reagent was used to access to cell viability. This test is based on cellular environment which reduces resazurin to resorufin. Viable cells increased resorufin in the medium. And resorufin, which is fluorescent, can be measured. The assays were conducted as described the manufacturer’s protocol. First, 90 µL of fresh medium was replaced in each plate. Then, 10 µL of PrestoBlue^TM^ HS Cell Viability Reagent per well were added. The plate was incubated for 1 h at 37 °C. Then, the medium was transferred in a black 96-well plate (Perkin Elmer Optiplate 96-F blake Polystyrene 6005270, Waltham, MA, USA). Measure of fluorescence was assessed with the spectrofluorometer Horiba Fluoromax-4 and plate reader Micromax, with the excitation wavelength at 560 nm and emission at 590 nm.

#### 2.3.2. Observation of Spheroid’s Morphologies

The morphology of the spheroids was monitored over 18 days using an optical microscope. Observations were made on Day 0, D1, D4, D7, D11, D14, and D18. Photographs were captured using a camera: v2512 (Phantom, Wayne, NJ, USA) from D0 and D1, and Orca Fusion (Hamamatsu, Hamamatsu City, Japan) from D4 to D18.

#### 2.3.3. Gemcitabine Sensitivity

To evaluate the sensitivity of these spheroids compared to a standard chemotherapeutic treatment, the spheroids were exposed to various concentrations of gemcitabine hydrochloride (Sigma-Aldrich G6423-10 mg) after 4 days of co-culture. At day 4 of spheroid formation, the culture medium was replaced with 100 µL of a gemcitabine solution at a 0, 0.3, 1, 3, 9, or 27 mM concentration. The gemcitabine solution was replaced once after 4 days of incubation. The viability of spheroids for the gemcitabine treatment were evaluated by CellTiter-Glo™ 3D Cell Viability Assay after a total of 7 days of incubation with gemcitabine.

### 2.4. Statistical Analysis

All data were analyzed using GraphPad PRISM v.10.0 software, presented as mean values ± standard deviation (SD). All the data sets were tested with a normality test by PRISM, and then statistical significance between different groups was analyzed using the ANOVA test with Bonferroni multiple comparisons correction. The statistical differences were considered significant when the *p*-value was lower than 0.05.

## 3. Results

### 3.1. CT3D Assays and Flow Cytometry

**Figure 3:**
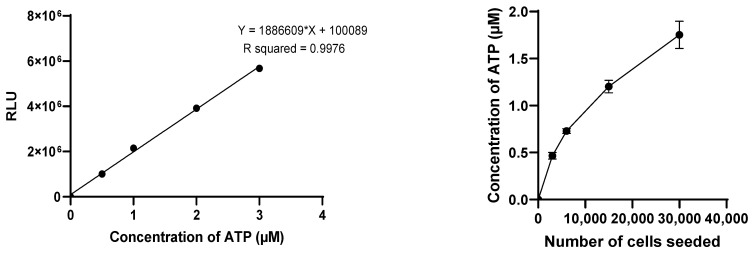
Linear regression of ATP (**left**). Viability of SphM with different number of cells seeded by CT3D assay. N = 3 per assay (**right**).

Figure 3 shows the ATP production of SphM, assessed with CT3D assay. Initial total cell counts of 3000, 6000, 15,000, and 30,000 cells were evaluated. The viability of these spheroids was measured after 11 days of incubation (D11). Assays demonstrated a non-linear increase in the intensity of luminescence with the number of seeded cells seeded. The ATP measurement was validated by a linear regression.

**Figure 4:**
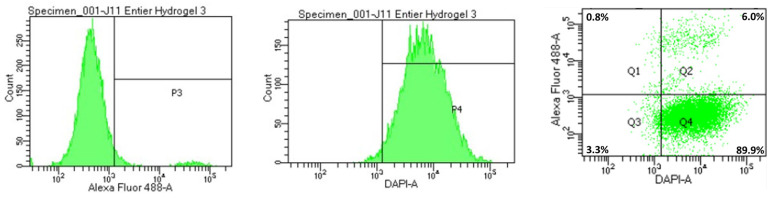
Detection of EGFP (**left**) and calcein (**middle**) in KPC and iMEF cells. (The horizontal line on the figures appears arbitrarily when you look at a population. It does not provide any information.) Distribution of cells according to the detection of EGFP and calcein (**right**). This gives us the percentages of live IMEFs and KPC A219. This example is from the dissociation of 8 SphG at D11.

Figure 4 shows an example of flow cytometry assay. The left panel shows the detection of EGFP-positive iMEF cells in the KPC and iMEF co-cultures using the Alexa Fluor channel (P3 window). The rest of the cell population represents KPC cells. In the middle panel, calcein-blue, as a marker of viable cells, was detected in the DAPI channel. Viable cells were visible in the P4 window. The graph on the right shows the distribution of cells according to the detection of the two fluorochromes, representing the proportion of live iMEFs and KPC A219.

### 3.2. Spheroids Viability

**Figure 5:**
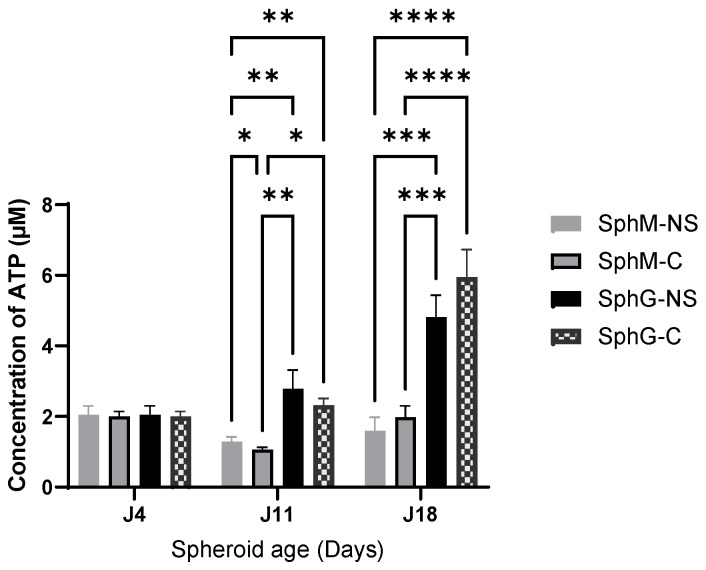
Viability of SphM and SphG (created with nanoshuttle (NS) or by centrifugation (c)) along with incubation time presented in ATP concentration. * difference is statistically significant, *p* < 0.05; ** difference is statistically significant, *p* < 0.01; *** difference is statistically significant, *p* < 0.001; **** difference is statistically significant, *p* < 0.001. Average ± standard deviation (SD). N = 6.

Figure 5 shows the ATP concentration measured with the CT3D assay as a function of the spheroid incubation period. The data reveal a continuous decline in the viability of SphM with time, whereas for SphG, the viability dropped at D4 but then increased back to its initial level of day 1.

Nanoshuttles do not change the viability measurements compared to spheroid formation by centrifugation. The rest of the spheroid characterization was carried out only on spheroids formed with nanoshuttles. This method has already been used in a previous study [27].

In addition, Figure S1 shows the ATP concentration measured with the CT3D assay as a function of the spheroid incubation period. The data reveal a continuous decline in the viability of SphM with time, whereas for SphG, the viability dropped at D4 but then increased back to its initial level of day 1.

**Figure 6:**
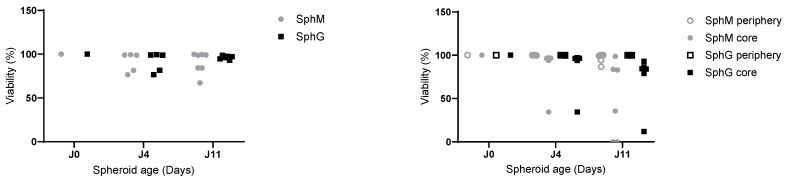
Normalized viability of whole (**left**) or dissociated (**right**) SphM or SphG at 4 and 11 days of incubation time. N = 6 for each experiment.

Figure 6 shows the viability of the spheroids’ peripheries and cores measured by flow cytometry (living cells were calcein-positive). The core viability was higher in spheroids cultured in hydrogel compared to that of spheroids cultured in medium. Only one case of necrotic core was observed in hydrogel spheroids, at D11.

### 3.3. Spheroids Morphology

**Figure 7:**
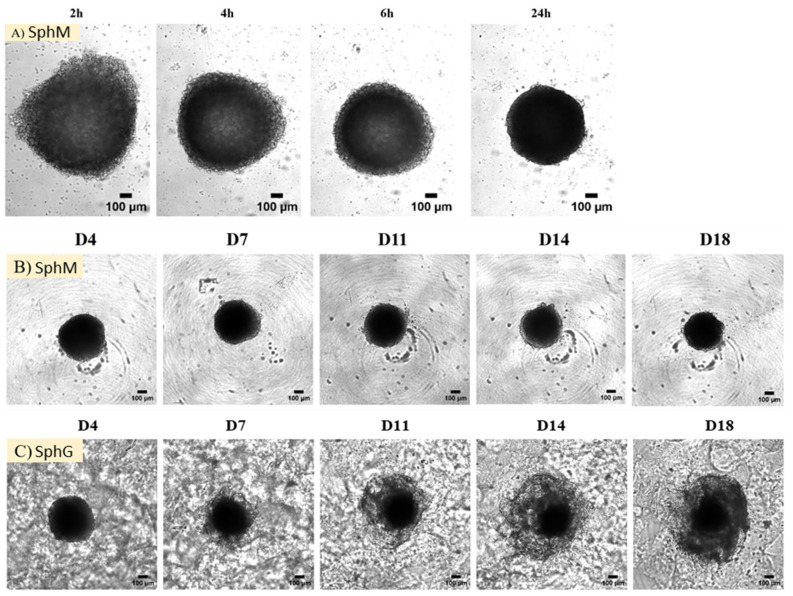
Morphology of the spheroids along with the incubation time. (**A**) Generation of spheroids in the culture medium during 24 h; (**B**) morphology of the spheroids incubated in the culture medium during 2 weeks from day 4 to day 18; (**C**) morphology of the spheroids cultured in the GrewDex gel during 2 weeks from day 4 to day 18. Scale bar: 100 µm.

**Figure 8:**
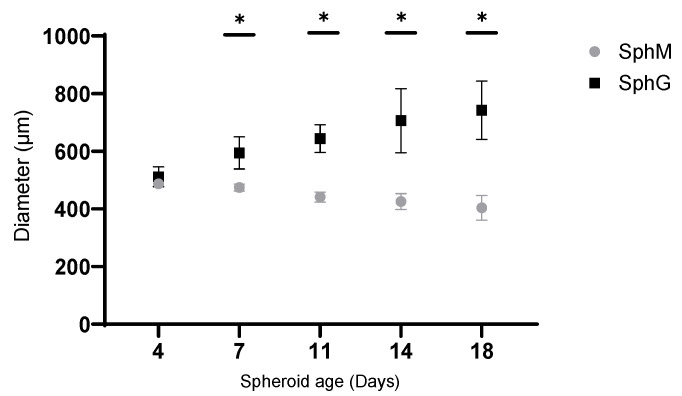
Diameter of SphM and SphG with the incubation time during 2 weeks from D4 to D18. * difference is statistically significant, *p* < 0.05. Average ± SD, N = 6.

Figure 7A shows the formation of spheroids over a 24 h span. Over the incubation period, there was a gradual aggregation of the cells. Initial observations of the spheroid transparency suggest that the cell aggregation formed a single layer at the beginning and took on a spheroidal shape with a concave center at the end of the first 6 h. Following 24 h incubation, the spheroids had a more consistent spherical shape, characterized by a distinct, darkened center. At the same time, the size of spheroids decreased with the incubation time. The diameter of spheroids after 24 h was around 630 µm. As incubation continued, the size of SphM decreased about 20% (500 µm) at D4. Following 2 weeks of incubation from D4 to D18, the size decreased slightly with the incubation time (Figure 7B and Figure 8). The morphology of SphM did not change significantly within the timeframe of observation.

Contrary to the SphM, a transparent structure, distinct from D7, formed around the main body of SphG and seemed to extend outward over time (Figure 7C and Figure 8). At the same time, the dark center part of spheroid seemed to migrate towards the transparent structure. This gave the impression that the SphG was growing. The diameter of SphG increased by 40%, up to around 740 µm at D18 compared to that of D4.

### 3.4. Composition of Spheroids

**Figure 9:**
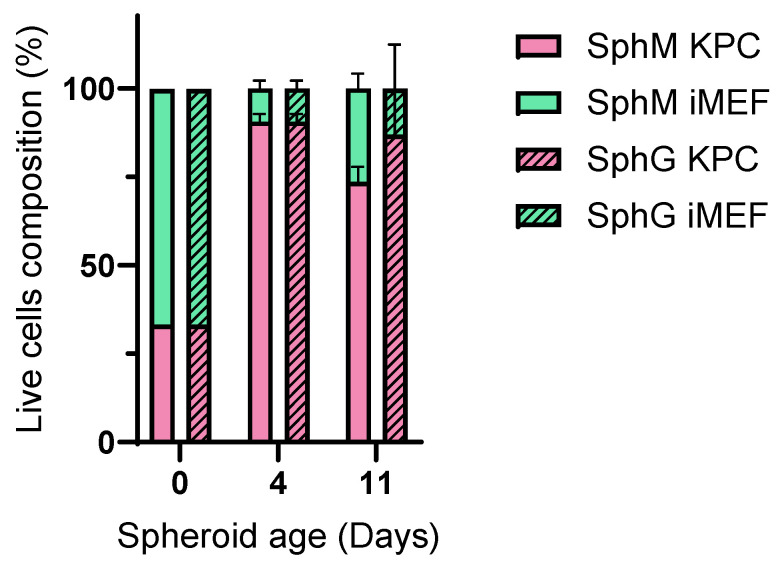
Percentage of iMEFs or KPC A219 in SphG and SphM. N = 7.

Figure 9 shows the composition of SphM and SphG in terms of the percentage of iMEF cells versus KPC A219 cells. Despite the two-to-one ratio of iMEF to KPC, the KPC cells rapidly outgrew the iMEF so that at D4, the iMEF-KPC ratio was only one-to-nine.

### 3.5. Gemcitabine Sensitivity

**Figure 10:**
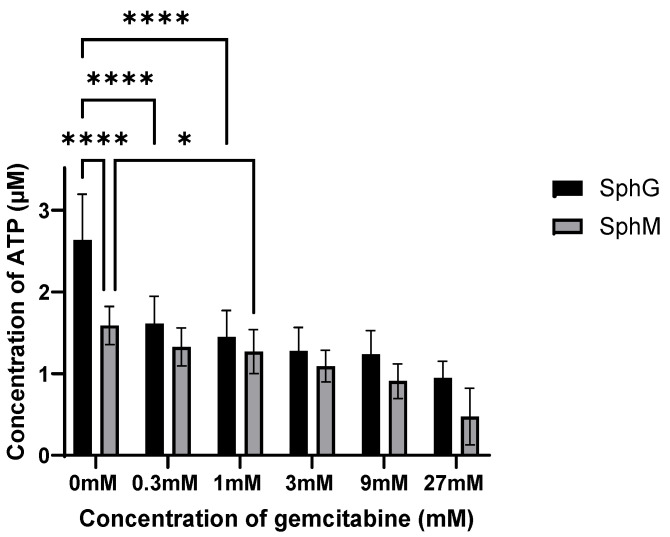
Viability of SphM and SphG after 11 days of gemcitabine incubation at various concentrations. * difference is statistically significant, *p* < 0.05; **** difference is statistically significant, *p* < 0.0001. Average ± SD. N = 10 minimum per groups.

Figure 10 shows the concentration of ATP produced by the spheroids (indicative of their viability) exposed to gemcitabine at a concentration ranging from 0 to 27 mM. Viability declined concomitantly with the concentrations of gemcitabine in the assessed range.

**Figure 11:**
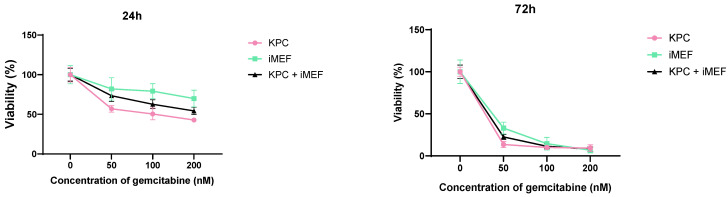
Viability of iMEFs versus KPC cells in 2D culture measured with Prestoblue after 24 or 72 h of incubation. N = 5.

**Figure 12:**
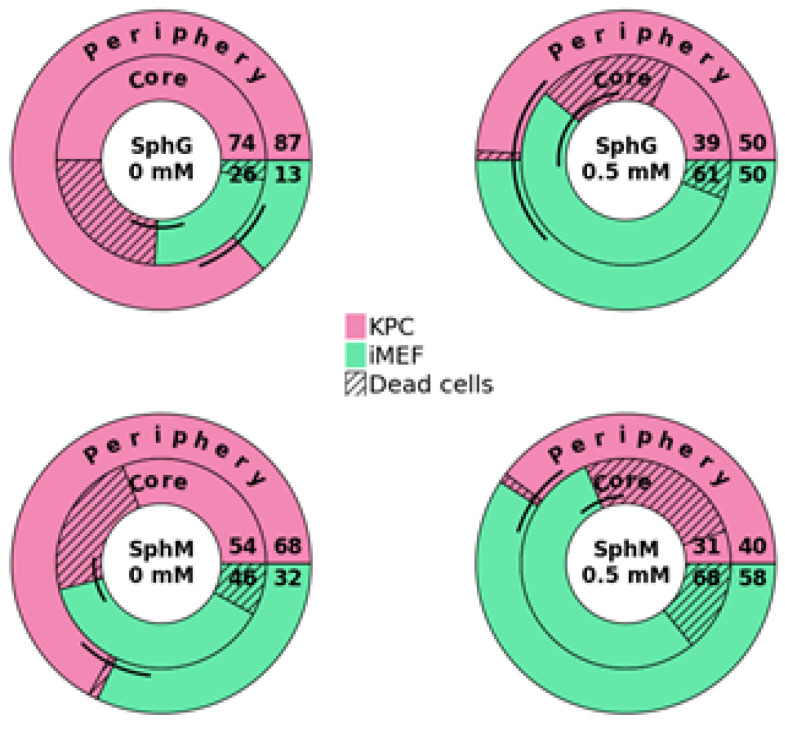
Percentage of iMEFs or KPC A219 in SphG and SphM as a function of gemcitabine concentration. The black arc represents the error margin. N = 12.

Figure 11 and Figure 12 show the difference in sensitivity of iMEFs and KPC cells to gemcitabine in 2D versus 3D. In the spheroids, when the KPCs (which are more sensitive to gemcitabine) died, the iMEFS remultiplied. In the periphery of the spheroid, iMEFs and KPCs still had very good viability. Dead KPC cells were evacuated from the spheroid and replaced by iMEFs. In the core of the spheroid, a higher proportion of dead KPC cells than iMEFs was observed under all conditions. This test was performed with 0.5 mM gemcitabine. Indeed, Figure 10 shows that SphG viability decreased significantly with 0.3 mM gemcitabine, whereas 1 mM was required for SphM.

## 4. Discussion

In this study, we applied the magnetic 3D bioprinting technology to generate the spheroids by co-culturing the KPC A219 pancreatic tumor cells and iMEF fibroblasts. The generation of spheroids under 24 h validated the method. The main advantage of this method is that it gives spheroids the characteristic of being magnetic while being simple and reproducible. The observation of SphM morphology under optical microscope showed that the spheroids diameter declined by 20% in the first 4 days incubation and then remained stable for up to 2 weeks. This initial shrinking has also been reported by other groups [20,30] and has been attributed to strong cell–cell and cell–ECM interactions [25,30]. Consequently, excluding the first 4 days, the spheroids produced in our study were stable in size and viability from D4 to D15, enabling the evaluation of cytotoxic agents within this timeframe. Morphologic observations and viability measurements throughout a 2-week period showed that contrarily to the SphM, the SphG continued to grow and to maintain viability. Following viability measurements carried out by flow cytometry, cell viability in the core of SphG was high than in SphM. We assume that this was due to a more favorable cell–ECM interaction with the hydrogel. The GDT hydrogel was compatible with the magnetic PDAC spheroids’ formation and favorable for spheroid growth. This growth was attributed to the formation of a less dense structure around the initial SphG, which we did not observe on the spheroids cultured in culture medium. This structure was likely composed of the ECM components and the proliferating or migrating cancer cells that formed a proliferative zone [31,32,33]. We have not performed a study to correlate spheroid size and viability in the spheroid core. Thanks to our viability measurements carried out by flow cytometry, we were able to observe better cell viability in the heart of SphGs. We assume that this was due to a more favorable cell–ECM interaction with the hydrogel. This feature made the spheroids more representative of the in vivo PDAC tumor environment.

The combination of the magnetic 3D bioprinting technique and hydrogel scaffold technique is an efficient method to obtain viable and growing spheroids, as pointed by Bowser and Moore [33]. They used the magnetic 3D bioprinting technique to produce magnetic 3D spinal cord spheroids and then incubated them into a synthetic hydrogel construct able to provide external guidance and direct the neurite projections. In our case, the spheroid formation with magnetic nanoparticles provided batch-to-batch reproducibility in shape and size. The cellulose-based hydrogel provided a support for the development of ECM environment that could mimic in vivo settings and allow the analysis of cell adhesion and migration.

The cell viability assays are pivotal for spheroid characterization and evaluating the cytotoxicity of drugs. Accurate results hinge on choosing a cell viability assay that is congruent with the cellular models utilized. Although 2D cells models remain predominant in biological testing today, their respective viability assay reagents continue to be a prevalent choice for evaluating 3D cells’ model viabilities [22,23]. However, with the development of 3D culture and characterization tools, reagents specifically designed for 3D cell viability assays have been developed. Numerous studies have indicated that the CT3D assay demonstrates greater sensitivity compared to similar methods [34,35,36]. The CT3D method also allows comparison of the spheroid viability along time, which is not possible with the flow cytometry analysis. However, particularly in spheroids with a dense collagen stroma, measured ATP can be influenced by both cell viability and CT3D penetration into the spheroid. Nonetheless, we observed that our spheroids were sensitive to gemcitabine. This suggests sensitivity to clinically relevant drugs, therefore validating this model.

Measuring viability using a flow cytometer allowed to differentiate the cell types present in the spheroid and to measure viability at the heart of the spheroid. This additional level of analysis provides more insight on the effect of a particular drug, notably its ability to penetrate the stroma. This method requires pooling eight spheroids, which makes it rather time- and resource-consuming. Considering the advantages and drawback of these two methods, we suggest that spheroid culture in medium with CT3D is cheaper, easier, and allows for an efficient first approach before refining one’s study with hydrogel-cultured spheroids with flow cytometry analysis. The cost/time ratio must be studied for each study in order to determine the best method to use. Hydrogel requires an additional cost but allows better analysis of viability in the core of the spheroid.

Measuring viability using a flow cytometer allowed us to determine that from day 4, iMEFs only represented 10% of the cells. Their presence was essential at the beginning of the spheroid formation, but very quickly, the KPC A219 became more numerous.

While our study provides a valuable insight into the PDAC spheroids’ generation method through the comparison of two different spheroids models, it is important to acknowledge the limitations that may affect the interpretation of our results. These limitations include the unique cell number seeded for the formation of spheroids that can affect results. Spheroids with diameters between 200 and 500 µm develop chemical gradients such as oxygen concentration, nutrients, and catabolites. For spheroids larger than 500 µm, necrosis in the core of the spheroid is expected [31,32]. The characterization of our spheroids models is limited in their overall characteristics. More microscopic assessments could be conducted, such as the evaluation of the composition of the transparent structure of SphG by marking the proteins such as collagen, one of the important components in ECM. For more detailed characterization, a gene and protein expression study would be useful. We did not evaluate whether the fibroblasts were contributing to extracellular matrix production, growth factor secretion, or immune modulation within the spheroids, which would constitute an interesting perspective for this study. The distribution of viable or dead cells inside of spheroids and in different spheroids’ ages to evaluate their proliferation and migration state could also be imaged.

Despite these constraints, our study contributes to the development of PDAC 3D models and may enable the optimization of future PDAC 3D models by integrating the magnetic 3D bioprinting technique with the hydrogel scaffold system. Future studies should aim to address these limitations for a more comprehensive understanding of PDAC spheroids’ characteristics for further improvement.

## 5. Conclusions

We developed and presented two types of PDAC spheroids in free culture or in hydrogels as well as two viability assessment methods. Results revealed that these models are complementary and could be used sequentially: spheroids culture in medium induces more variability but is less expensive and easier than hydrogel culture. Likewise, ATP production measurement is fast, cost-effective, and could be performed at different timepoints, ideal for a first approach, but lacks specificity and could be influenced by penetration into the spheroids. Flow cytometry solves this issue while providing information on the spheroid periphery compared to its core, at the cost of the heavy experimental constraint that a large number of spheroids are needed.

## Data Availability

The data presented in this study are available on request from the corresponding author, pending publication.

## Supplementary Materials

The following supporting information can be downloaded at: https://www.mdpi.com/article/10.3390/mps8040075/s1, Figure S1: ATP concentration measured with the CT3D assay.
