# Supplementary figures and images for: Formation and Characterization of Two Magnetic Three-Dimensional Spheroid Models of Murine Pancreatic Adenocarcinoma
Source: Methods Protoc. 2025 Jul 7;8(4):75. doi: 10.3390/mps8040075 (PMC12286017; doi:10.3390/mps8040075)

**Figure S1:** ATP concentration measured with the CT3D assay.

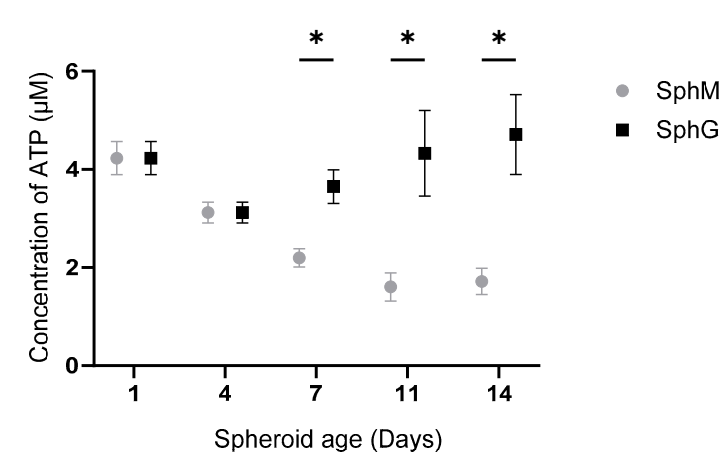

Supplement: Supplementary file 1 [file mps-08-00075-s001.zip › mps-3695037-supplementary.pdf]
